# Emotional aging: a discrete emotions perspective

**DOI:** 10.3389/fpsyg.2014.00380

**Published:** 2014-05-06

**Authors:** Ute Kunzmann, Cathleen Kappes, Carsten Wrosch

**Affiliations:** ^1^Institute of Psychology, University of LeipzigLeipzig, Germany; ^2^Department of Psychology, Concordia UniversityMontreal, QC, Canada

**Keywords:** emotional aging, adult lifespan, anger, sadness, affective well-being

## Abstract

Perhaps the most important single finding in the field of emotional aging has been that the overall quality of affective experience steadily improves during adulthood and can be maintained into old age. Recent lifespan developmental theories have provided motivation- and experience-based explanations for this phenomenon. These theories suggest that, as individuals grow older, they become increasingly motivated and able to regulate their emotions, which could result in reduced negativity and enhanced positivity. The objective of this paper is to expand existing theories and empirical research on emotional aging by presenting a discrete emotions perspective. To illustrate the usefulness of this approach, we focus on a discussion of the literature examining age differences in anger and sadness. These two negative emotions have typically been subsumed under the singular concept of negative affect. From a discrete emotions perspective, however, they are highly distinct and show multidirectional age differences. We propose that such contrasting age differences in specific negative emotions have important implications for our understanding of long-term patterns of affective well-being across the adult lifespan.

The single most important finding in the field of emotional aging is perhaps that affective well-being does not decline during most of the adult lifespan (e.g., Charles and Carstensen, 2007; Scheibe and Carstensen, 2010; Isaacowitz and Blanchard-Fields, 2012). Research based on the most prominent life-span theories of developmental regulation and emotional aging have provided different explanations for this phenomenon by examining broad dimensions of emotions (i.e., negative affect and positive affect; e.g., Baltes and Baltes, 1990; Brandtstädter and Greve, 1994; Heckhausen, 1997; Carstensen, 2006). Complementing these theories, we present a discrete emotions perspective, that considers specific positive and negative emotions. To illustrate the usefulness of this approach, we focus on a discussion of age differences in the experience of anger and sadness. These two negative emotions have typically been subsumed under the singular concept of negative affect. From a discrete emotions perspective, however, they are highly distinct and show multidirectional age differences. We propose that such contrasting age differences in specific emotions have important implications for understanding long-term patterns of affective well-being across the adult lifespan.

## Life-span theories of developmental regulation and emotional aging

Prominent theories of developmental regulation postulate that individuals can avoid the adverse emotional effects of life's challenges, threats, and losses if they engage in effective self-regulatory strategies (e.g., Baltes and Baltes, 1990; Brandtstädter and Renner, 1990; Heckhausen et al., 2010). Although the nature of strategies has been defined and labeled differently by different theories, the various strategies seem to cluster according to whether they refer to continued engagement in attaining goals or to disengagement from unattainable goals (Haase et al., 2013). Further, the extent to which these two higher-order categories of developmental regulation protect affective well-being is thought to differ as a function of an individual's place in the life-cycle, which can be considered a proxy for individual differences in control opportunities and constraints. Given that individuals' capacity to influence their environment is relatively high in young adulthood and often declines in old age, processes of goal engagement should be used more frequently and adaptive in young adulthood. Processes of goal disengagement, by contrast, should become increasingly salient and adaptive in older adulthood. A large number of longitudinal and intervention studies have supported these propositions (reviews: Wrosch et al., 2006; Heckhausen et al., 2010).

Socioemotional Selectivity Theory (SST; Carstensen, 2006; Scheibe and Carstensen, 2010; Reed and Carstensen, 2012), a prominent theory of emotional aging, explains the maintenance of affective well-being in old age by focusing on future-related expectations, particularly older individuals' estimations of their remaining life-time. According to SST, because advancing age is naturally associated with endings and a limited lifetime, older adults prioritize social and emotional goals related to the optimization of immediate affective well-being over longer-term knowledge-related goals aimed at optimizing future resources (Carstensen et al., 1999). In support of SST's main predictions, a large body of evidence suggests that, in comparison with their younger counterparts, older adults are more selective in their choice of social partners and make their social interactions more emotionally satisfying (reviews: Carstensen et al., 1999; Fingerman and Charles, 2010). There is also substantial evidence for systematic age differences in basic affective information processing. Older adults appear to be generally more sensitive to positive information and less sensitive to negative information than young adults, a phenomenon termed the “positivity effect” (Carstensen and Mikels, 2005). SST states that this positivity effect is the result of an age-related increase in the allocation of cognitive resources toward emotion regulation (reviews: Mather and Carstensen, 2005; Kryla-Lighthall and Mather, 2009; Mather, 2012).

Overall, life-span developmental theories have provided valuable insights into how older individuals can maintain or enhance their affective well-being despite age-related losses in many life domains. Independent of their specific explanatory processes, however, affective well-being has been considered in these theories from a broad and dimensional perspective. That is, research based on these theories typically examined whether processes of developmental regulation, socio-emotional selectivity, or biases in affective information processing are associated with a broader aggregate of emotional experiences such as positive affect or negative affect. Past theoretical work is thus well suited to explain age differences in broad affective dimensions, but it may fall short in explaining age-differences in the salience and functions of different discrete negative or positive emotions subsumed under positive or positive affect. As a consequence, a discrete emotions perspective may be needed to complement past theoretical work. Such an approach may help better understand multidirectional age differences in short-term affective reactions and longer-term affective well-being. In addition, it may raise awareness that emotions are not only consequences of cognitive or motivational processes, but also can cause effective cognitions and behaviors.

## A discrete emotions perspective on emotional aging

Functional emotion theories emphasize the adaptive value of discrete emotions and emotional dispositions (e.g., Frijda, 1986; Campos et al., 1989; Lazarus, 1991; Izard, 1993; Levenson, 1994; Ekman, 1999; Keltner and Gross, 1999). According to these theories, emotions provide important information to the self and others and help motivate our own and others' behavior. For example, some emotions push individuals away from certain thoughts, memories, or actions, whereas other emotions make certain thoughts, memories, or actions more likely. Functional approaches traditionally have focused on the adaptive value of negative emotions. From this perspective, the overall purpose of negative emotions is to eliminate a threat or an imbalance between the individual and his or her environment. More specifically, negative emotions are thought to prompt time-tested adaptive actions enabled and supported by specific action tendencies (Frijda, 1986), patterns of physiological activity (Levenson, 1994), configurations of facial expressions (Ekman, 1999), and subjective feelings (e.g., Izard, 1993). Functional approaches to emotion have emphasized that each discrete negative emotion serves a specific and distinct function (e.g., fear creates the urge to escape, disgust the urge to expel; e.g., Frijda, 1986; Levenson, 1992).

### Age differences in negative affect: the sample case of anger and sadness

To explore the usefulness of a discrete emotion approach, we focus on the functions of two emotions, anger and sadness for two reasons. First, empirical studies examining age differences in discrete emotions have largely focused on these two emotions. Second, and more important, different from other discrete emotions, anger and sadness are closely and differentially associated with the two higher-order classes of developmental regulation, goal engagement and goal disengagement. As a consequence, a theoretical framework that addresses age differences in the salience and adaptivity of anger and sadness appears to be a reasonable starting point for developing a more comprehensive discrete emotions approach.

A central tenet of theories of life-span development has been that each stage in the life cycle is characterized by a specific configuration of environmental challenges as well as personal needs, beliefs, and future expectations (e.g., Heckhausen and Schulz, 1995). We build on this assumption and argue that age-specific configurations may increase or decrease the experience and adaptive value of specific emotions (see also Haase et al., 2012). This proposition provides a theoretically important extension of functional emotion theories as it suggests that not only the emotion itself but also characteristics of the individual and his or her immediate and broader social context, most of which substantially change across the adult life-span, determine the adaptive value of specific emotions.

To begin, young adulthood has been described as a phase of growth during which individuals have greater opportunities to develop their potentials than their older counterparts. Thus, processes of growth and optimization rather than maintenance or compensation should have priority in young adulthood (Baltes and Baltes, 1990; Freund and Baltes, 1998, 2002; Ebner et al., 2006). Consistent with this view, SST assumes that young, as compared with older adults' goals tend to be more future-oriented and focused on acquiring new resources such as knowledge or information (e.g., Carstensen et al., 1999). Moreover, life-span theories of developmental regulation (e.g., Brandtstädter and Greve, 1994; Heckhausen et al., 2010) have proposed that, in young adulthood, perceptions of high personal control and a tenacious pursuit of goals are not only highly prevalent, but also closely tied to well-being (Wrosch and Heckhausen, 1999; Rothermund and Brandtstädter, 2003; Lachman, 2006). Finally, there is evidence suggesting that individuals are most willing to engage in and reflect on social conflicts when they are young (Labouvie-Vief et al., 1989; Birditt and Fingerman, 2005; Blanchard-Fields et al., 2007).

Given that young adults typically pursue many future-related goals and have a strong need to accomplish them, the elicitor of anger, which is the appraisal that one's own goals are intentionally ignored or blocked by others, should be particularly frequent and salient in young adulthood. In addition, the situational control appraisals, action tendencies, and social motivations typical of anger are likely to be more readily accessible in young adulthood than in old age. In fact, according to discrete emotion theories, anger prototypically involves the belief in high situational control (Lazarus, 1991), triggers a reactant “moving against” state of action readiness (Frijda et al., 1989), and promotes goal persistence (Lench and Levine, 2008). In addition, anger facilitates efforts to bring others' behaviors back in line with one's own goals (Fischer and Roseman, 2007). Thus, relative to older adults, young adults should experience anger more intensively and more frequently. Moreover, because anger may at times help young adults to overcome obstacles in accomplishing age-normative tasks, it could be adaptive, at least in certain contexts, and benefit young adults' longer-term affective well-being (Haase et al., 2012).

In marked contrast to young adulthood, old age has been characterized as a phase of loss and decline (Lindenberger and Baltes, 1997; Smith and Baltes, 1997; Baltes and Mayer, 1999) during which social, cognitive, and physical resources become increasingly limited and processes of maintenance and compensation gain importance (Baltes and Baltes, 1990; Labouvie-Vief, 2003). Life-span theories have proposed that, given the limited resources in old age, perceptions of low personal control and goal adjustment processes become increasingly frequent and adaptive (e.g., Wrosch and Heckhausen, 1999; Rothermund and Brandtstädter, 2003; Lachman, 2006). In addition, according to SST, because perceived future life-time shrinks with increasing age, older adults' tend to be particularly present-oriented in their goal pursuits, focused on creating emotionally meaningful experiences, and likely to avoid social conflicts for the sake of maintaining social harmony in the current moment (Birditt and Fingerman, 2005; Carstensen, 2006; Blanchard-Fields, 2007).

Given that, relative to young adults, older adults increasingly face losses of fundamental resources and tend to adjust their goals accordingly, the elicitor of sadness, that is, the appraisal of a situation as an irreversible loss, should be particularly frequent and salient in old age. In addition, the situational control appraisals, action tendencies, and social motivations that are linked to sadness are likely to be more readily accessible in old age than in earlier life periods. In support of this assumption, discrete emotion theories suggest that sadness prototypically goes hand in hand with a perception of low situational control (Lazarus, 1991). In addition, it triggers processes of adaptive goal disengagement (Frijda et al., 1989; Nesse, 2000; Wrosch and Miller, 2009) and signals to others the need for closeness, sympathy, and support (Andrews and Thomson, 2009). As a consequence, relative to young adults, older adults should experience sadness more intensively and frequently. Moreover, because sadness is likely to help older adults deal with losses by facilitating disengagement from unattainable goals and enhancing social support, it could be adaptive, at least in certain contexts, and facilitate older adults' longer-term affective well-being (see also Kunzmann and Grühn, 2005; Haase et al., 2012).

### Empirical evidence

A small but growing body of evidence is consistent with the notion that sadness and anger follow contrasting trajectories across the adult life-span. For example, research has shown that older adults report less anger and anger-related regulatory strategies during interpersonal conflicts than their younger counterparts (Blanchard-Fields and Coats, 2008). An age-related reduction in the intensity of anger reactivity was also found when young and older adults were confronted with film clips of an old woman talking about problems in a nursing home (Charles et al., 2001) or audio-taped conversations, in which two individuals were ostensibly making disparaging remarks about a third person (Charles and Carstensen, 2008). Age differences in sadness-reactions, by contrast, have been shown to be reversed (i.e., higher among older adults) or non-significant (Charles et al., 2001; Charles and Carstensen, 2008). Other studies have investigated age differences in emotional reactions to stimuli specifically designed to elicit either anger or sadness. In this line of work, older, as compared with younger, adults reported less anger in response to anger-eliciting stimuli, but equal or higher sadness in response to sadness-eliciting stimuli (Tsai et al., 2000; Labouvie-Vief et al., 2003; Kunzmann and Grühn, 2005; Kunzmann and Richter, 2009; Seider et al., 2011; Streubel and Kunzmann, 2011).

Age-related differences in the experience of anger and sadness have also been examined on the level of affect frequency in a longitudinal field study with a national sample of German adults covering most of the adult lifespan (Kunzmann et al., 2013). More specifically, cross-sectional and longitudinal findings from this study consistently suggest that the frequency of anger increases from late adolescence into young adulthood, but shows a steady decrease from midlife to old age. By contrast, the frequency of sadness remained stable over most of adulthood and increased in old age (Kunzmann et al., 2013). Extending this work, a recent study explored age differences in anger and sadness by focusing on the intensity and frequency of emotional experiences on a typical day and their implicit associations with participants' self (Kunzmann and Thomas, 2014). Consistent with a discrete emotions perspective, older adults experienced anger less frequently and less intensively than young adults, but there were no age differences in the experience of sadness. Furthermore, this study provided initial evidence for the idea that anger, but not sadness, becomes less self-descriptive with age (Kunzmann and Thomas, 2014).

Finally, there is preliminary evidence indicating that the effects of anger and sadness on affective well-being are age-differential as well (Haase et al., 2012). In this laboratory study, anger experiences in response to neutral film clips with ambiguous contents were associated with high affective well-being in middle-aged adults but not in young or older adults. Sadness reactions, by contrast, were associated with high affective well-being in older adults, but not in younger or middle-aged adults (Haase et al., 2012). These findings lend support to our previous predictions, although according to our framework, anger should be adaptive in young adulthood as well.

## Conclusions and outlook

Life-span theories' current focus on the explanation of age-related changes in the overall quality of affective experience does not account well for multidirectional and multifunctional age differences in specific emotions. A discrete emotions perspective on aging therefore complements life-span theories as it allows more differentiated predictions. According to this view, all emotions—positive and negative—are adaptive in particular contexts. Thus, although most individuals value high emotional well-being (Tsai et al., 2006), and report pro-hedonic emotion regulatory goals more frequently than contra-hedonic goals (Riediger et al., 2009), from a functionalist view, a life without negative emotions would be impoverished, unhealthy, and short. Instead, it assumes that negative emotions are useful experiences. They serve specific functions and may be adaptive if they enable individuals to manage critical life circumstances.

Our approach and review is consistent with this view by documenting the usefulness and importance of a discrete emotions approach. It demonstrates multidirectional age differences in the experience of anger and sadness by indicating that the salience of anger decreases across the life-span, particularly during middle adulthood and old age, whereas the intensity and frequency of sadness remains stable or increases in old age. In addition, it provides preliminary evidence suggesting such age differences in emotional reactions may contribute to individuals' general affective well-being.

To further clarify the roles of specific negative emotions, future research should determine the conditions that give rise to adaptive consequences of anger reactions (in young adulthood) and sadness reactions (in old age). Certainly, chronic or unwarranted anger and sadness are unlikely to contribute to high affective well-being at any age. Instead, it may be the match between situational circumstances and emotional reactions that could determine the adaptive value of age-related experiences of anger and sadness. To examine such situation by emotion interactions, future research should also incorporate time windows of different lengths because specific negative emotions may not be strongly associated with high levels of concurrent affective well-being. Rather, the experience of certain negative emotions could restore and optimize individuals' affective well-being over longer periods of time if they trigger and facilitate behavioral processes that over time help individuals to adjust successfully to age-related opportunities and constraints.

A second task for future work is to shed further light on the mechanisms that can explain age differences in anger and sadness. Our theoretical framework is motivational in nature in that we argue that each place in the life-cycle is characterized by a specific configuration of developmental conditions that make certain emotions more salient and adaptive. However, theoretical accounts of age differences in cognitive and physiological resources may provide additional insights (Labouvie-Vief, 2003; Cacioppo et al., 2011). For example, anger may involve greater physiological arousal and may require more cognitive resources than sadness, and this may imply that older adults have fewer resources to experience anger than sadness.

Finally, it will be interesting to extend our framework by considering additional distinct negative and positive emotions. For example, there is preliminary research suggesting age-related increases in fear and age-related declines in disgust (Kunzmann et al., 2005; Teachman and Gordon, 2009). Future research should clarify the mechanisms that underlie such age differences in a variety of distinct emotions. Research along these lines may also broaden the age range of inquiry by additionally examining children and adolescents and consider the influence of individual differences in available resources and types of goals. We think that future research from a discrete emotion perspective is warranted and has the potential to contribute to our understanding of successful development across the lifespan.

## Author note

The completion of this manuscript was supported by grants from the German Research Foundation to Ute Kunzmann and by grants from Social Sciences and Humanities Research Council of Canada and Canadian Institutes of Health Research to Carsten Wrosch.

### Conflict of interest statement

The authors declare that the research was conducted in the absence of any commercial or financial relationships that could be construed as a potential conflict of interest.
